# International Ovarian Tumor Analysis Scoring and Its Histopathological Correlation: A Diagnostic Accuracy Study

**DOI:** 10.7759/cureus.103593

**Published:** 2026-02-14

**Authors:** Dhanya Paul, Alphonse Thomas

**Affiliations:** 1 Obstetrics and Gynaecology, Monsignor Uralil Memorial (MUM) Hospital, Monippally, IND; 2 Community Medicine, Sarojini Naidu Medical College, Agra, IND

**Keywords:** diagnostic accuracy, histopathology, iota simple rules, ovarian tumors, ultrasonography

## Abstract

Background: Accurate preoperative differentiation between benign and malignant ovarian tumors is essential for optimal patient management and surgical planning. Ultrasonography remains the first-line imaging modality, and the International Ovarian Tumor Analysis (IOTA) Simple Rules provide a standardized approach for risk stratification.

Objective: This study aimed to evaluate the diagnostic accuracy of IOTA scoring in differentiating benign and malignant ovarian tumors by correlating ultrasound findings with histopathological outcomes.

Methods: This prospective diagnostic validation study included 75 women with ovarian tumors undergoing surgical management at the Department of Obstetrics and Gynaecology, Institute of Maternal and Child Health (IMCH), Government Medical College, a tertiary care teaching hospital in Kozhikode, India, between January and December 2019. Preoperative ultrasonography was performed using transabdominal and/or transvaginal approaches, and ovarian masses were classified according to the IOTA Simple Rules. Histopathology served as the reference standard. Sensitivity, specificity, positive predictive value (PPV), and negative predictive value (NPV) were calculated.

Results: Abdominal pain was the most common presenting symptom (62.7%). Histopathology confirmed malignancy in 9.3% of cases, with benign tumors comprising the majority. Epithelial tumors represented the most common histopathological subtype (88%). Among benign ultrasound features, acoustic shadowing (B3) was the most frequent, while unilocular cysts (B1) demonstrated the highest predictive accuracy. Among malignant features, ascites (M2) showed the highest predictive performance. IOTA scoring demonstrated a sensitivity of 85.7%, specificity of 91.2%, PPV of 50.0%, and NPV of 98.4%.

Conclusion: The IOTA Simple Rules demonstrate good diagnostic accuracy in the preoperative evaluation of ovarian tumors. The particularly high negative predictive value supports their utility as a reliable, non-invasive triage tool for safely excluding malignancy in benign-dominant populations, thereby aiding appropriate surgical planning and referral decisions

## Introduction

Ovarian tumors are commonly encountered in gynecological practice and encompass a wide spectrum of benign and malignant conditions. Differentiating benign from malignant ovarian masses prior to surgery is a critical clinical challenge, as it directly influences surgical planning, referral to specialized oncology services, and overall patient prognosis. Ovarian cancer remains one of the leading causes of gynecologic cancer-related mortality worldwide, largely because the disease is frequently diagnosed at an advanced stage due to nonspecific early symptoms [1,2].

Ultrasonography is the preferred first-line imaging modality for the evaluation of adnexal masses because it is noninvasive, widely available, and cost-effective [3]. Despite these advantages, conventional ultrasound interpretation is highly dependent on the examiner’s experience, leading to variability in diagnostic accuracy [4]. To improve objectivity and standardization, several diagnostic models have been developed, including morphological scoring systems, Doppler flow analysis, and the Risk of Malignancy Index (RMI), which combines ultrasound findings with serum CA-125 levels [5,6]. However, these models may be complex and show variable performance across different clinical settings.

The International Ovarian Tumour Analysis (IOTA) group introduced the Simple Rules, a standardized ultrasound-based classification system using five benign and five malignant features to evaluate ovarian masses [7]. The Simple Rules were designed to be easy to apply, reproducible, and effective across varying levels of operator expertise. Multiple validation studies have demonstrated high sensitivity and acceptable specificity of this system in distinguishing benign from malignant adnexal masses [8,9].

Accurate preoperative identification of malignant ovarian tumors is essential, as improved survival outcomes have been reported when surgical management is performed by trained gynecologic oncologists [10]. The present study evaluates the diagnostic performance of the IOTA Simple Rules in differentiating benign and malignant ovarian tumors by correlating preoperative ultrasound findings with histopathological results.

## Materials and methods

Study design

This study was designed as a diagnostic test validation study.

Study setting

The study was conducted in the Department of Obstetrics and Gynaecology, Institute of Maternal and Child Health (IMCH), Government Medical College, Kozhikode, India.

Study period

The study was carried out over a period of one year, from January 1, 2019, to December 31, 2019.

Study population

The study population included patients with ovarian tumors undergoing surgery in the Department of Obstetrics and Gynaecology, IMCH, Kozhikode.

Sampling procedure

All eligible patients who presented during the study period and met the inclusion criteria were recruited using consecutive sampling.

Sample size calculation

Sample size calculation was based on a study by Kaijser J et al. [11], which reported a specificity of 81% for the IOTA Simple Rules. The prevalence of malignant ovarian tumors in the previous year in our department was 21%.

The minimum sample size was calculated using the formula:

Where: Zα/2 = 1.96 for α = 5%

d = 10%

The calculated minimum sample size was 74.8, which was rounded off to 75 patients.

Inclusion criteria

Patients presenting with ovarian tumors, either unilateral or bilateral, within the age group of 16 to 80 years, who were undergoing surgical treatment, were included in the study. In cases of bilateral ovarian tumors, the lesion with the most complex ultrasound morphology was included. If bilateral tumors demonstrated similar ultrasound morphology, the largest lesion or the lesion that was most easily accessible by ultrasonography was selected for evaluation.

Exclusion criteria

Pregnant patients with ovarian tumors were excluded from the study. Patients undergoing emergency surgery for an acute abdomen and those who had received neoadjuvant chemotherapy were also excluded.

Methodology

A total of 75 patients with ovarian tumors who satisfied the inclusion and exclusion criteria and attended the Department of Obstetrics and Gynaecology for surgical treatment were selected. Data were collected using a pre-designed proforma after obtaining informed written consent from all patients.

A detailed history was obtained, including presenting complaints, duration of symptoms, menstrual history, parity, oral contraceptive pill usage, dietary history, history of ovarian, breast, or other tumors, and family history of malignancy. All patients underwent thorough clinical examination, including general physical examination, systemic examination, abdominal examination, and bimanual pelvic examination, to arrive at a clinical diagnosis of ovarian tumor. Further investigations were carried out to confirm the diagnosis.

Ultrasonography, including transabdominal or transvaginal sonography with Doppler, was performed, and sonological features were analyzed by an experienced sonologist in the Department of Radiodiagnosis, Government Medical College, Kozhikode. The IOTA Simple Rules, proposed by the International Ovarian Tumor Analysis (IOTA) group, were applied to assess the likelihood of malignancy in ovarian tumors. Timmerman et al., 2008 [8].

Tumors were classified according to the International Ovarian Tumor Analysis (IOTA) Simple Rules [8]. A tumor was considered benign when at least one benign (B) ultrasound feature was present in the absence of any malignant (M) features. A tumor was classified as malignant when at least one malignant (M) feature was identified without the presence of benign features. In cases where both benign and malignant features were present, or when no rule was applicable, tumors were categorized as malignant for analytical purposes to avoid underestimation of malignancy risk.

Contrast-enhanced computed tomography (CECT) was performed to evaluate tumor extension, lymph node metastasis, soft tissue involvement, ascites, and upper abdominal disease. Tumor markers, including Cancer Antigen-125 (CA-125) and Carcinoembryonic Antigen (CEA), were assessed along with routine blood investigations. A CA-125 value of <35 IU/L was considered normal, while values >35 IU/L were considered abnormal. A CEA value of <2.5 ng/ml was considered normal, and values >2.5 ng/ml were considered abnormal.

All patients underwent surgery as the primary treatment modality. Patients with benign features underwent laparotomy with unilateral ovarian cystectomy or ovariotomy, or laparoscopy with unilateral ovarian cystectomy. Patients with malignant features underwent staging laparotomy, which included total abdominal hysterectomy with bilateral salpingo-oophorectomy and infracolic omentectomy. Intraoperative findings suggestive of malignancy, such as ascites, multilocularity, solid components, and a number of papillary projections, were recorded.

The excised specimens were sent for histopathological examination to the Department of Pathology, Government Medical College, Kozhikode. Histopathological evaluation was performed by pathologists who were blinded to the ultrasound findings. Sociodemographic factors were analyzed for their association with ovarian malignancy. Histopathological reports were correlated with IOTA scoring, CECT findings, and intraoperative findings. Histopathological evaluation also included assessment for tubal involvement, specifically serous tubal intraepithelial carcinoma (STIC).

Patients diagnosed histopathologically with ovarian cancer (stage >1C) were referred to the Department of Radiation Oncology, Government Medical College, Kozhikode, for adjuvant chemotherapy and were followed up after completion of six cycles of chemotherapy. Patients with benign pathology (stage <1C) were followed up six-monthly with clinical examination and ultrasonography.

Data analysis

Data were entered into Microsoft Excel (Microsoft Corp., USA) and analyzed using IBM SPSS Statistics for Windows, Version 18.0 (released 2009, IBM Corp., Armonk, NY) for Windows. Qualitative data were expressed as frequencies and percentages, while quantitative data were expressed as mean or median with standard deviation. Statistical analysis was performed using Fisher’s exact test and Student’s t-test. A p-value of less than 0.05 was considered statistically significant.

Ethical issues

The study was approved by the Institutional Ethics Committee of Government Medical College Kozhikode, with approval number GMCKKD/RP 2019/IEC/90. Written informed consent was obtained from all patients included in the study.

## Results

Table 1 presents the clinical profile and tumor characteristics of the study population (N = 75). The majority of patients were aged 40-60 years (35, 46.7%), followed by those aged 20-40 years (27, 36.0%). Patients aged 60-80 years accounted for nine (12.0%) cases, while four (5.3%) were younger than 20 years.

**Table 1:** Clinical profile and tumor characteristics of the study population

Variable	n (%)
Age (years)	
<20	4 (5.3%)
20–40	27 (36.0%)
40–60	35 (46.7%)
60–80	9 (12.0%)
Presenting Symptoms	
Abdominal pain	47 (62.7%)
Abdominal distension	12 (16.0%)
Other symptoms	16 (21.3%)
Tumor size	
<5 cm	18 (24.0%)
5–10 cm	28 (37.3%)
10–20 cm	22 (29.3%)
>20 cm	7 (9.3%)
Tumor consistency	
Cystic mass	55 (73.3%)
Solid mass	3 (4.0%)
Solid–cystic mass	17 (22.7%)

Abdominal pain was the most common presenting symptom, reported in 47 (62.7%) patients. Abdominal distension was noted in 12 (16.0%) patients, while other symptoms were observed in 16 (21.3%) patients.

Regarding tumor size, most masses measured 5-10 cm (28, 37.3%), followed by 10-20 cm (22, 29.3%) and less than 5 cm (18, 24.0%). Tumors larger than 20 cm were seen in 7 (9.3%) patients.

On the assessment of tumor consistency, cystic masses were the most common type, observed in 55 (73.3%) patients. Solid-cystic masses were present in 17 (22.7%) patients, while purely solid masses were identified in three (4.0%) patients.

Table 2 presents the distribution and predictive accuracy of individual IOTA Simple Rule ultrasound features. 

**Table 2:** Distribution and predictive accuracy of the International Ovarian Tumor Analysis (IOTA) ultrasound features

IOTA feature	Predicted (n)	Result (n)	Percentage (%)
Benign features (B-rules)			
B1: Unilocular cyst	49	49	100.0
B2: Solid component <7 mm / absent	26	25	96.2
B3: Acoustic shadowing	64	63	98.4
B4: Smooth multilocular cyst <10 cm	14	13	92.9
B5: No blood flow	57	56	98.2
Malignant features (M-rules)			
M1: Irregular solid tumor	11	6	54.5
M2: Ascites	9	6	66.7
M3: ≥4 papillary projections	10	6	60.0
M4: Multilocular solid tumor ≥10 cm	12	6	50.0
M5: Very strong blood flow	10	6	60.0

Among the benign (B-rule) features, acoustic shadowing (B3) was the most frequently observed finding, identified in 64 cases, of which 63 were confirmed benign on histopathology (98.4%). Absence of blood flow (B5) was seen in 57 cases and correctly predicted benignity in 56 cases (98.2%). Unilocular cyst (B1) was observed in 49 cases and demonstrated 100% predictive accuracy. A solid component less than 7 mm or absent (B2) was present in 26 cases and correctly predicted benignity in 25 cases (96.2%). Smooth multilocular cysts measuring less than 10 cm (B4) were noted in 14 cases, with 13 confirmed benign (92.9%).

Among the malignant (M-rule) features, an irregular solid tumor (M1) was observed in 11 cases, of which six were confirmed malignant (54.5%). Ascites (M2) was present in nine cases and correctly predicted malignancy in six cases (66.7%), representing the highest predictive accuracy among malignant features. Papillary projections (≥4) (M3) and very strong blood flow (M5) were each identified in 10 cases and correctly predicted malignancy in 6 cases (60.0%). Multilocular solid tumors ≥10 cm (M4) were observed in 12 cases, with six confirmed malignant (50.0%).

Overall, benign IOTA features demonstrated high predictive accuracy, whereas malignant features showed comparatively moderate predictive performance.

Table 3 compares preoperative assessment modalities with histopathological findings, which served as the gold standard. 

**Table 3:** Comparison of preoperative assessment with histopathology IOTA: International Ovarian Tumor Analysis, CECT: contrast-enhanced computed tomography

Modality	Benign n (%)	Malignant n (%)
IOTA scoring	63 (84.0%)	12 (16.0%)
CECT findings	64 (85.3%)	11 (14.7%)
Intraoperative findings	64 (85.3%)	11 (14.7%)
Histopathology (gold standard)	68 (90.7%)	7 (9.3%)

IOTA scoring classified 63 (84.0%) cases as benign and 12 (16.0%) as malignant. CECT findings categorized 64 (85.3%) cases as benign and 11 (14.7%) as malignant. Intraoperative findings similarly identified 64 (85.3%) benign cases and 11 (14.7%) malignant cases.

Histopathological examination confirmed 68 (90.7%) benign cases and seven (9.3%) malignant cases. Preoperative modalities identified a marginally higher proportion of malignant cases compared with histopathological confirmation

Table 4 summarizes the diagnostic performance of IOTA scoring and tumor marker levels. 

**Table 4:** Diagnostic performance of IOTA scoring and tumor markers (N = 75) IOTA: International Ovarian Tumor Analysis, CEA: carcinoembryonic antigen

Parameter	Result
IOTA scoring – diagnostic performance	
Sensitivity	85.7%
Specificity	91.2%
Positive predictive value (PPV)	50.0%
Negative predictive value (NPV)	98.4%
CA-125 levels (IU/ml)	n (%)
<35	58 (77.3%)
35–100	8 (10.7%)
100–500	6 (8.0%)
>500	3 (4.0%)
CA-125 elevated	17 (22.7%)
CA-125 elevated in malignancy	5/7 (71.4%)
CEA levels (ng/ml)	n (%)
<2.5	62 (82.7%)
>2.5	13 (17.3%)
CEA elevated	13 (17.3%)
CEA elevated in malignancy	2/7 (28.6%)

IOTA scoring demonstrated a sensitivity of 85.7% and specificity of 91.2% in differentiating malignant from benign ovarian tumors. The positive predictive value was 50.0%, while the negative predictive value was 98.4%, indicating a strong ability to rule out malignancy when the test result was benign.

With respect to CA-125 levels, 58 (77.3%) patients had values <35 IU/ml. Levels between 35-100 IU/ml were observed in 8 (10.7%) patients, 100-500 IU/ml in 6 (8.0%), and >500 IU/ml in 3 (4.0%) patients. Overall, CA-125 elevation was noted in 17 (22.7%) cases. Among the histopathologically confirmed malignant cases, five out of seven (71.4%) demonstrated elevated CA-125 levels.

CEA levels were <2.5 ng/ml in 62 (82.7%) participants and >2.5 ng/ml in 13 (17.3%) participants. Elevated CEA levels were observed in 13 (17.3%) cases, among which two out of seven malignant cases (28.6%) showed elevation. 

## Discussion

In the present study, the majority of patients were in the 40-60-year age group, followed by those aged 20-40 years, with fewer cases at the extremes of age. Abdominal pain was the most common presenting symptom, and most adnexal masses were of moderate size (5-10 cm) with predominantly cystic morphology. This clinical profile reflects a largely benign disease spectrum, which is typical of adnexal masses presenting for surgical evaluation in tertiary care settings.

This distribution is comparable to the original IOTA development study by Timmerman et al. [8], in which most adnexal tumors were benign and predominantly cystic. Similar clinical patterns have been described in studies evaluating adnexal masses using standardized ultrasound-based risk stratification systems [11]. The systematic review and meta-analysis by Gareeballah et al. [12], which included 7,841 adnexal masses, demonstrated a broad clinical spectrum, with pain and mass-related symptoms being common presenting features. Differences in clinical presentation across studies are largely attributable to variations in inclusion criteria and underlying malignancy prevalence.

In our cohort, histopathological examination confirmed malignancy in 9.3% of cases, indicating a benign-dominant population. The IOTA Simple Rules demonstrated good diagnostic performance, with a sensitivity of 85.7% and specificity of 91.2%. The high specificity suggests that the system was effective in correctly identifying benign lesions, thereby potentially reducing unnecessary aggressive surgical management.

A particularly notable finding was the very high negative predictive value (98.4%), reflecting the strong ability of IOTA scoring to reliably exclude malignancy when a benign classification was assigned. In low-prevalence settings such as ours, negative predictive value becomes especially important for clinical reassurance and safe conservative management. The positive predictive value, however, was moderate (50.0%), which can be explained by the low prevalence of malignancy in the study population. Predictive values are inherently influenced by disease prevalence; therefore, in benign-dominant cohorts, PPV tends to decrease despite good sensitivity and specificity. This finding underscores the importance of interpreting predictive values within the clinical context.

Evaluation of individual IOTA features revealed that benign rules demonstrated excellent predictive performance. Acoustic shadowing (B3), absence of blood flow (B5), and unilocular cysts (B1) showed very high accuracy in predicting benign pathology. Among malignant features, ascites demonstrated the highest predictive accuracy, although overall malignant rule performance was moderate. This asymmetry likely reflects the biological heterogeneity of malignant and borderline ovarian tumors and highlights the strong discriminatory value of benign rules when present.

The diagnostic performance of the IOTA Simple Rules has been validated across diverse populations. Sayasneh et al. [13] demonstrated reliable performance of IOTA prediction models across operators with varying expertise. Feharsal and Putra [14] similarly reported good diagnostic performance of the IOTA scoring system in predicting ovarian malignancy preoperatively, further supporting its applicability in routine clinical practice. Sharma et al. [15] also demonstrated a strong correlation between IOTA classification and histopathological findings, reinforcing the reliability of structured ultrasound assessment. Nunes et al. [16] confirmed stable diagnostic accuracy across multiple clinical settings, supporting reproducibility. Dakhly et al. [17] reported high sensitivity but variable specificity in cohorts with higher malignant prevalence, illustrating how performance metrics may shift depending on case mix. Alcázar et al. [18] further validated the IOTA Simple Rules in a prospective external study, confirming high sensitivity in differentiating benign from malignant adnexal masses.

Preoperative IOTA scoring, contrast-enhanced computed tomography, and intraoperative findings slightly overestimated malignancy compared with histopathology in our study. This cautious tendency may reflect an intentional bias toward minimizing missed malignant cases, which is clinically acceptable in oncologic evaluation.

Tumor markers provided adjunctive value. CA-125 was elevated in 71.4% of malignant cases but also showed elevation in some benign conditions, consistent with its known lack of specificity. CEA demonstrated limited diagnostic utility, with elevation observed in only 28.6% of malignant cases. These findings reinforce that tumor markers should complement, rather than replace, structured ultrasound assessment.

Overall, the present study supports the use of the IOTA Simple Rules as a reliable and practical tool for preoperative evaluation of adnexal masses. In benign-dominant populations, the system provides high confidence in ruling out malignancy, while malignant features warrant careful interpretation and correlation with clinical and biochemical findings.

Limitations of the study 

Despite its strengths, this study has several limitations. The sample size was relatively small (N = 75), which may limit generalizability and reduce the precision of diagnostic performance estimates, particularly the positive predictive value. The study was conducted at a single tertiary care center, introducing potential referral bias. The low prevalence of malignancy (9.3%) influenced predictive values and limited detailed subgroup analysis of malignant features. Inter-observer variability in ultrasound interpretation was not formally assessed. In addition, long-term follow-up of conservatively managed benign lesions was not included.

## Conclusions

Ovarian tumors in this study were most commonly observed in women aged 40-60 years, with abdominal pain as the predominant presenting symptom, and the majority demonstrating cystic morphology. The IOTA Simple Rules showed good diagnostic accuracy in differentiating benign from malignant ovarian tumors, with particularly high specificity and an excellent negative predictive value. This highlights their reliability as a non-invasive triage tool for safely excluding malignancy in benign-dominant populations.

While the positive predictive value was moderate, likely due to the low prevalence of malignancy, malignant features warrant careful interpretation in conjunction with clinical findings and tumor markers. Elevated CA-125 levels were frequently associated with malignancy, whereas CEA demonstrated limited diagnostic utility.

Overall, the integration of IOTA scoring with clinical assessment and CA-125 estimation provides an effective and practical framework for preoperative risk stratification and surgical planning in patients with ovarian tumors.
